# Speckle tracking echocardiography-derived left ventricular global longitudinal strain in ex-thalassaemics

**DOI:** 10.1371/journal.pone.0293452

**Published:** 2023-11-01

**Authors:** Amal Paul, Uday Kulkarni, Bijesh Yadav, Fouzia N. Aboobacker, Anup J. Devasia, Anu Korula, Aby Abraham, Biju George, Paul V. George, Alok Srivastava

**Affiliations:** 1 Department of Cardiology, Christian Medical College Vellore, Ranipet Campus, Tamil Nadu, India; 2 Department of Haematology, Christian Medical College Vellore, Ranipet Campus, Tamil Nadu, India; 3 Department of Biostatistics, Christian Medical College, Vellore, Tamil Nadu, India; Cairo University Kasr Alainy Faculty of Medicine, EGYPT

## Abstract

**Aims:**

Long term survivors of haematopoietic stem cell transplantation (HSCT) for β-thalassemia major are designated “ex-thalassaemics”. Whether ex-thalassaemics continue to harbour residual myocardial dysfunction and thereby stand the risk of heart failure-related morbidity and mortality is unknown. The aim of this study was to assess the prevalence and predictors of subclinical left ventricular (LV) dysfunction in an apparently normal ex-thalassaemic population.

**Methods:**

We conducted a single centre cross-sectional study among 62 ex-thalassaemic patients, who had undergone HSCT for β-thalassaemia major at our centre. The primary outcome variable was LV systolic dysfunction, as assessed by 1) LV global longitudinal strain (GLS) derived by 2D speckle tracking echocardiography and 2) LV ejection fraction (EF) derived by 2D Simpsons Biplane method.

**Results:**

Among the 62 patients included in the study, 7 [11.3%] were found to have LV systolic dysfunction, all of which were subclinical. Of these, 4 [6.5%] had abnormal GLS and LVEF, 2 [3.2%] had abnormal GLS with normal LVEF, and 1 [1.6%] had abnormal LVEF with low normal mean GLS. There were no statistically significant predictors of LV dysfunction in this cohort.

**Conclusion:**

There was a high prevalence of subclinical myocardial dysfunction in the ex-thalassaemic population reiterating the need for close follow up of these patients. 2D Speckle tracking echocardiography-derived LV global longitudinal strain is an effective tool in detecting subclinical myocardial dysfunction in this cohort.

## Introduction

Heart disease accounts for almost three quarters of deaths in patients with β-thalassaemia major [1]. The prevalence of heart failure reported in thalassaemic patients with a mean age of 27 years was 2.5% in 2004 [2]. The outcomes of these patients have substantially improved over the past decades [3]. The 5-year survival rate of thalassaemics after the onset of heart failure was reported to be 48% in 2001 [4], as compared to the 3-month survival rate of less than 50% in the 1960s [5].

Allogeneic haematopoietic stem cell transplantation [HSCT] has revolutionized the treatment of beta-thalassaemia major as a curative option [6]. Thalassaemic patients who have undergone HSCT as a curative therapy are designated as “ex-thalassaemics”. Whether ex-thalassaemics continue to harbor residual myocardial dysfunction and thereby stand the risk of heart failure-related morbidity and mortality during their survivorship has not been studied till date. A better understanding of cardiac function in these patients is therefore needed [4].

At present, magnetic resonance imaging [MRI] scan is considered to be the investigation of choice for detection of iron overload in patients with beta thalassaemia. MRI assessment of cardiac T2* can identify and quantify cardiac iron stores, thus identifying those individuals at risk of developing cardiac complications [3, 7, 8]. However, repeated cardiac MRI evaluation is often not feasible for monitoring of these patients due to limited access to the technology and other logistic difficulties in the paediatric population. There are also challenges pertaining to calibration and quality control of cardiac MRI in these patients [9].

Myocardial strain, which is the relative deformation of myocardium during cardiac cycle, as assessed by 2D speckle tracking echocardiography, has been demonstrated to be a sensitive tool in detecting subclinical left ventricular [LV] dysfunction in patients with various cardiomyopathies including chemotherapy-induced LV dysfunction and iron overload cardiomyopathy, with minimal inter- and intra-observer variability [10–14]. In patients with heart failure and improved LVEF, an abnormal LV GLS has been found to predict the likelihood of subsequent deterioration of LVEF, underscoring the prognostic significance of LV GLS in apparently normal patients [15].

The aim of this study was to assess the prevalence and predictors of subclinical LV dysfunction in an apparently normal asymptomatic ex-thalassaemic population.

## Patients and methods

We conducted a single centre cross-sectional prospective study on ex-thalassaemic patients. All ex-thalassaemic patients, who had undergone HSCT for β-thalassaemia major at our centre, and came for follow-up between March and September 2018, and were willing for cardiac evaluation were screened and included in the study. Exclusion criteria included history of documented congenital or acquired heart disease in the past. The study was approved by the institutional review board (IRB:11134) and the ethics committee of our institution. Informed written consent and assent were obtained from the participants or their parents. Investigators had access to information that could identify individual participants.

The conditioning regimen for HSCT was determined based on Pesaro risk stratification, which classifies patients with thalassaemia major into three groups (class I, II and III), based on liver size (> 2 cm), presence of liver fibrosis, and inadequate chelation. Higher pre-transplant Pesaro class, which reflects a greater burden of iron overload, has been shown to be associated with worse clinical outcomes following HSCT [16]. The conditioning regimen consisted of intravenous Busulfan started at 0.8 mg per kg body weight per dose every 6 hours for 4 days with therapeutic drug monitoring-guided dose adjustment (from day -9 to day -6) along with intravenous Cyclophosphamide 50 mg per kg body weight per day for 4 days (day -5 to day -2), and equine Anti-thymocyte globulin (ATGAM) at 30 mg per kg body weight per day for 3 days (from day -8 to day -6) for patients with Pesaro class I and II risk status [16]. The conditioning regimen for patients with Pesaro class III risk status consisted of intravenous Thiotepa administered at 8 mg per kg body weight for 1 day (day -6), intravenous Treosulfan 14 g per m^2^ body surface area per day for 3 days (from day -5 to day -3), and intravenous Fludarabine 40 mg per m^2^ body surface area per day for 4 days (from day -5 to day -2). Graft Versus Host Disease [GVHD] prophylaxis consisted of Cyclosporine and short course Methotrexate in all patients as previously reported [17] except for one patient who received post-transplant Cyclophosphamide along with Cyclosporine as part of an ongoing clinical trial. Exclusion criteria included pre-existing congenital or acquired cardiac disease.

All patients were evaluated with two dimensional [2D] echocardiography by the same investigator, who was blinded to the haematological profile of the patients. Global longitudinal strain [GLS] was determined by 2D speckle tracking echocardiography in all these patients, using Philips Epiq 7 machine (Q lab software version 1.5.2). The primary outcome variable was LV systolic dysfunction evidenced by diminished 2D speckle tracking echocardiography-derived left ventricular global longitudinal strain [LVGLS] or LV ejection fraction [LVEF] derived by Simpson’s biplane method. The cut-off for LVGLS was accepted as -19.5% based on standard recommendations [18, 19], as this value has also been shown to correlate with significant myocardial iron deposition in thalassaemia patients across all age groups [20, 21].

### Statistical analysis

Acquired data were summarized as mean (standard deviation, SD) for continuous variables, and percentages for categorical variables. Continuous variables were compared using t-test, and categorical variables with chi-square/Fisher’s exact test. Predictors of LV dysfunction were analyzed using penalized regression analysis, and expressed as Odds Ratio (OR) with 95% confidence intervals (CI) for continuous variables and absolute risk (AR) with 95% CI for categorical variables. Statistical significance was defined as P<0.05. All analyses were done using SPSS v25 and SAS 9.4.

## Results

### Clinical profile

Sixty-two patients were included in the study for analysis. The median age was 8 years [range: 2–22]. There were 40 [64.5%] males. The median age at the time of HSCT was 7 years [range: 1–17]. As per the Pesaro risk stratification for HSCT for thalassaemia major [16], 3 were class I, 17 were class II, and the remaining 42 were class III, of which 18 were deemed to be Vellore high risk category [17], prior to HSCT.

The median time elapsed between HSCT and the echocardiographic study was 13 months [range: 1–72 months]. While most patients did not receive any potentially cardiotoxic drugs during HSCT, 19.3% [n = 12] of the patients had received cyclophosphamide (cumulative dose of 200 mg/kg) as a constituent of the conditioning regime. There were no cases of documented cardiac toxicity during or following HSCT. None of the patients had clinical or laboratory documented evidence of heart failure either at the time of the study or in the past.

### Iron overload

The median number of red cell transfusions till the date of echocardiographic study in the study group was 80 [range: 10–500]. The median time elapsed since the last transfusion was 13 months [range: 1–72]. The median of peak serum ferritin levels documented in the study group was 3187 ng/mL [range: 303–9811]. The median of serum ferritin levels in the study group at the time of echocardiographic evaluation was 1981 ng/mL [range: 24–9811].

Iron reduction therapy was administered to 56 [90.3%] patients, with either oral [n = 46] or parenteral [n = 10] chelating agents, as per institutional protocols, with the goal of reducing serum ferritin levels to <300 ng/mL. Out of these, 10 patients were still on chelation therapy at the time of the study. The median duration of chelation therapy was 53 months in the study group [range: 6–120 months].

### Cardiac function

Left ventricular systolic function was assessed by Simpsons 2D LV ejection fraction [LVEF] as well as 2D LV GLS. The mean LVEF was 59.7 +/- 5.4% in the study group. 92% of the patients [n = 57] had normal LVEF. The rest had mild LV systolic dysfunction. None had LVEF <45%.

The mean LV GLS was -22.4 +/- 2.3% in the study group. 9.7% of the patients [n = 6] had LV systolic dysfunction, with abnormal LVGLS. It was noted that an additional 13% [n = 8] had abnormal LV GLS in atleast one of the three imaging planes [namely apical four-chamber (AP4), three-chamber (AP3), or two-chamber (AP2) views], although the mean GLS was normal.

Overall, 9.7% [n = 6] of the participants had abnormal mean LV GLS and 8.1% [n = 5] had abnormal LVEF. Among the 5 patients with reduced LVEF, 4 were found to have reduced mean LV GLS as well. The remaining one patient had low normal mean GLS but with an abnormal GLS in one of the imaging planes. Thus 11.3% [n = 7] of the study group was found to have myocardial dysfunction, all of which were subclinical.

Diastolic function was found to be normal in all the patients studied. There was no evidence of pulmonary hypertension or right ventricular systolic dysfunction in any of the patients. Apart from subclinical LV systolic dysfunction, no other abnormality was detected in the echocardiographic study. The profile of patients, who were detected to have LV dysfunction, is depicted in **Table 1**.

**Table 1:** Clinical and haematological profile of patients with LV dysfunction.

Age [Years]	Sex	Pesaro class	Pesaro-Vellore class	Number of transfusions	Duration of chelation [Months]	Age at HSCT [years]	Conditioning regimen	Months post transplant [at the time of the study]	Pre-transplant Ferritin [ng/mL]	Peak Ferritin [ng/mL]	Ferritin at the time of the study [ng/mL]
6	M	III	IIILR	80	72	6	TTF	3	6250.7	6250.7	8007
7	M	III	IIILR	40	102	4	TTF	37	1600	2699	361.5
8	M	II	II	60	72	7	TTF	12	660.8	754	867
8	M	III	IIIHR	80	72	7	TTF	6	2678.1	2739	4232
14	F	III	IIILR	180	96	14	TTF	1	110.5	263	230.9
15	M	III	IIIHR	290	96	15	TTF	5	3876.3	5881	4080
22	F	II	II	300	96	22	TTF	3	3154	3338	4523

### Predictors of LV dysfunction

Indices of iron overload such as pre-transplant serum Ferritin values, pre-transplant Pesaro class, or evidence of iron overload on MRI (cardiac T2* or hepatic T2* done prior to HSCT) did not correlate with subclinical LV dysfunction. The results of logistic regression analysis are depicted in **Table 2**.

**Table 2:** Correlation of various factors with LV dysfunction.

Variables	LV dysfunction (N = 7)	Normal LV function (N = 55)	OR (95% CI) & AR (95% CI)	p Value
Age at study enrolment (years, mean ± SD)	11.43±5.83	9.44±4.92	1.08(0.93–1.24)	0.31
Male sex (n, %)	5(71.4)	35(63.6)	3.41 (-12.38, 19.20)	0.69
Number of transfusions (mean ± SD)	147.14±110.3	108.87±95.2	1.00 (0.99–1.01)	0.26
Duration of chelation(months, mean ± SD)	76.29±11.34	77.22±21.42	0.99 (0.96–1.04)	0.95
Age at the time of HSCT (years, mean ± SD)	10.71±6.47	7.62±4.64	1.12 (0.97–1.29)	0.12
Time since HSCT (months, mean ± SD)	9.57±12.59	20.93±19.39	0.96 (0.89–1.04)	0.29
Pre-transplant ferritin (ng/mL, mean ± SD)	2618±2090	3064±1999	0.99 (0.99–1.00)	0.74
Pre-transplant Cardiac T2* (n, %) 0 1 2 3	5(83.3) 0(0) 0(0) 1(16.7)	23(56.1) 7(17.1) 7(17.1) 4(9.8)	1.00 -17.86 (- 32.04, - 3.67) -17.86 (- 32.04, - 3.67) 2.14 (- 35.68, 39.97)	0.24 0.24 0.91
Pre-transplant Hepatic T2* (n, %) 0 1 2 3	1(16.7) 2(33.3) 2(33.3) 1(16.7)	9(20.9) 12(27.9) 13(30.2) 9(20.9)	1.00 4.29 (- 21.82, 30.40) 3.33 (- 222.0, 28.66) -	0.76 0.81 -
Pesaro class (n, %) 0 1 2	0(0) 2(28.6) 5(71.4)	3(5.5) 15(27.3) 37(67.3)	1.00 11.76 (- 3.55, 27.08) 15.63 (3.0, 28.21)	0.54 0.47
Pesaro-Vellore class (n, %) 0 1 2 3	0(0) 2(28.6) 3(42.9) 2(28.6)	3(5.5) 15(27.3) 21(38.2) 16(29.1)	1.00 11.76 (- 3.55, 27.08 12.50 (- 0.73, 25.73) 11.11 (- 3.41, 25.63)	0.54 0.52 0.55

**Note:** OR = odds ratio & AR = Absolute risk

Interestingly, none of the twelve patients who received Cyclophosphamide-based conditioning regimen prior to HSCT were found to have LV systolic dysfunction. In other words, all patients who were noted to have LV systolic dysfunction on echocardiography, had received a non-cardiotoxic conditioning regime consisting of Thiotepa, Treosulphan and Fludarabine (TTF).

For the 7 patients with subclinical LV dysfunction, the median ferritin around the time of GLS testing was 4080 ng/mL (interquartile range: 867 to 4523). At the last follow up of these patients, the median ferritin was 2000 ng/mL (interquartile range: 185.3 to 3282). Four patients still had ferritin above 1000 ng/mL. However, all had normal effort tolerance for their daily activities.

## Discussion

With increasing success of HSCT for higher risk patients with thalassemia major and their long term survival, attention needs to be focused on potential organ dysfunction related to pre-HSCT complications. Iron overload and related organ damage are the most significant of these. There is limited data on cardiac function among ex-thalassemics.

This study is therefore significant as it evaluated a large number of ex-thalassaemics with no history of overt cardiac dysfunction over a wide age range (8–22 years) going into early adulthood and with long follow-up after HSCT. Given this profile, other risk factors for myocardial dysfunction were unlikely and none were identified in any of the patients studied, apart from cyclophosphamide based HSCT conditioning regimen which was found to have no correlation with the outcome. Our data shows that subclinical myocardial dysfunction exists in about 11% of ex-thalassemics. Further follow-up is needed to understand its evolution in time.

It has long been recognized that regular monitoring of myocardial function is warranted in patients with β-thalassaemia, especially in those receiving repeated blood transfusions, for early detection of subclinical myocardial dysfunction [22]. Although cardiac MRI is regarded as the gold standard in this regard, echocardiography remains the most readily available tool for evaluation and monitoring of myocardial dysfunction in these patients. Furthermore, pre-transplant T2* values did not correlate with LV dysfunction in the ex-thalassaemics. Our study confirms the utility of speckle tracking echocardiography for evaluation of LV dysfunction in this cohort. Results of a detailed literature review of studies reporting the efficacy of strain imaging in detecting myocardial dysfunction in patients with thalassaemia major is presented in **Table 3**.

**Table 3:** Summary of studies reporting the utility of strain echocardiography for evaluation of LV dysfunction among patients with thalassaemia major.

Study	Study modality and parameters	Salient findings	Inference
1. Attar et al. 2022 [20] Meta-analysis of data from 789 patients with Thalassaemia	Speckle tracking echocardiography [STE]- derived global longitudinal strain [GLS]	GLS <-19.5% predicts cardiac iron overload with 92.8% sensitivity and 34.63% specificity (AUC = 0.659, 95%CI = 0.6–0.72).	GLS is a strong predictor of cardiac iron overload, which may be useful as a screening method.
2. Abtahi F et al. 2019 [21] 52 patients with Thalassemia	Speckle tracking echocardiography [STE]- derived global longitudinal strain [GLS]	GLS <-19.5% could predict T2*level below 20 with 82.14% sensitivity and 86.36% specificity (AUC = 0.87, p<0.0001).	GLS may be used to screen thalassaemia patients for myocardial iron deposition, using a cut-off value of -19.5%.
3. Garceau et al. 2011 [23] 45 chronically transfused patients with thalassemia major or Diamond Blackfan anaemia, and 18 healthy controls	Speckle Tracking Echocardiography [STE]	Patients with low T2* had a uniform decrease in longitudinal and circumferential strain compared with normal controls. Global longitudinal strain [GLS] predicted a T2* value of <20 ms with a sensitivity of 76% and a specificity of 88%.	GLS offers a simple alternative to cardiac MRI for assessing significant myocardial iron deposition.
4. Ari et al. 2017 [14] 52 patients with thalassemia	Speckle Tracking Echocardiography [STE] and Tissue Doppler Imaging [TDI]	Septal systolic myocardial velocity and global strain (GLS) were significantly associated with cardiac MRI T2*	GLS may be used as a valuable marker to screen thalassemia patients for myocardial iron deposition
5. Narayana et al. 2017 [24] 25 patients with thalassemia and 25 healthy controls	Speckle tracking echocardiography	Patients had lower LVEF (62.20 ± 7.93% vs. 66.40 ± 1.19%, *P* < 0.001) and lower GLS (−21.73 ± 3.68% vs. −26.80 ± 1.29%, *P* < 0.001) as compared to the controls.	LVEF and GLS are lower even in young children with beta-thalassemia major
6. Rezaeian et al. 2020 [25] 91 patients with thalassemia and 23 healthy controls	Speckle tracking echocardiography	LV GLS, LV GCS, and right ventricular GLS had significant correlation with cardiac T2*	GLS may be used to detect myocardial iron overload
7. Abtahi et al. 2019 [11] 52 patients with thalassemia	Speckle Tracking Echocardiography [STE] and Tissue Doppler Imaging [TDI]	Septal systolic myocardial velocity and GLSwere significantly associated with T2*	GLS may facilitate the cardiac follow up in patients with thalassemia
8. Parsaee et al. 2017 [10] 55 patients with Thalassemia major	Speckle tracking echocardiography [STE]- derived global longitudinal strain [GLS]	There was a significant reduction in GLS. There was no significant difference in circumferential strain. There was no significant correlation between GLS and CMR T2 *.	STE-GLS is a feasible method for evaluating subclinical myocardial dysfunction in patients with thalassemia major, independent of CMR and extent of iron deposition.
9. Pizzino et al. 2017 [26] 28 patients with thalassemia	Speckle tracking echocardiography [STE]- derived global longitudinal strain [GLS]	Patients with impaired GLS (<-19.5%) had significantly higher myocardial iron overload [MIO]. No significant difference was found for radial and circumferential strain.	GLS can identify patients with severe MIO detected by CMR
10. Magrì et al. 2008 [27] 30 patients with thalassemia and 30 healthy controls	Tissue Doppler Imaging [TDI] and TDI-derived strain parameters	Systolic strain of RV free wall, septal wall, and lateral wall were significantly associated with T2*. RV EM, LV EM, septal EM, and septal SM showed a significant correlation with T2*.	TDI and TDI-derived strain parameters are both sensitive and specific in predicting presence of myocardial iron load in TM patients with normal LV global systolic function.
11. Hamdy 2007 [12] 27 patients with thalassemia and 14 controls	TDI and TDI-derived strain parameters	Systolic strain rate in LV, and septal-EM were lower in the study group. Sm and strain values were lower in the lateral LV wall compared to septal wall.	Strain imaging seems more capable of early detection of regional myocardial dysfunction.
11. Gupta et al. 2017 [28] 30 patients with thalassemia and 20 controls	TDI and TDI-derived strain parameters	Strain rate (SR) at the basal and mid segment of the lateral LV wall, and basal and mid septum; Lateral EM velocity were significantly lower in patients, as compared to controls.	PW-TDI, strain and SR imaging are useful to quantify regional myocardial function in asymptomatic β-TM patients with preserved global LV systolic function.
12. Silvilairat et al. 2008 [29] 100 thalassemia patients and 100 healthy controls	Tissue Doppler Imaging [TDI]	Basal septal Am, mid-septal Em and Am, basal lateral Am, mid-lateral LV wall Sm and Am were lower in the patient group	TDI can be used for screening of myocardial iron load in thalassemia major patients.
13. Vogel et al. 2003 [30] 52 thalassemia patients and 52 healthy controls	Tissue Doppler Imaging [TDI]	The incidence of wall motion abnormalities was significantly higher (P<0.04) in patients with myocardial iron overload (87%) than in the 14 without (35%)	The regional wall motion abnormalities, early sign of iron overload, are easily detectable with tissue Doppler echocardiography
14. Aypar et al. 2010 [31] 33 patients with thalassemia and 20 healthy controls	Tissue Doppler Imaging [TDI]	TDI measures such as sepal SM (Septal systolic myocardial velocity) and septal EM (Septal early diastolic myocardial velocity) showed a significant relationship with T2* Regional myocardial dysfunction was more prominent in patients with T2* ≤ 20 ms.	PW-TDI technique was found to be sensitive and specific in predicting presence of myocardial iron load in TM patients with normal LV global systolic function.

Iron reduction therapy implemented in ex-thalassaemics is often targeted at bringing serum ferritin to normal levels. Myocardial iron deposition may persist in such patients. In thalassaemia patients, this has been shown to be non-uniform in MRI-based studies, with different patterns of regional distribution [32, 33]. Pepe et al. reported a heterogeneous distribution of cardiac iron overload in about half of their patients with beta thalassemia major [32]. In their study, the mean serum ferritin value was found to be significantly higher in patients with heterogenous distribution of iron as compared to those with homogenous distribution [32]. Autopsy studies of iron distribution in hemochromatotic myocardium have also demonstrated marked heterogeneity of iron overload [34]. The exact mechanism behind this heterogeneity is not well established. The heterogeneity of myocardial iron distribution, which appears in patients with borderline iron overload seems to become more uniform at moderate to severe iron burden [35].

Heterogeneity has also been reported in resorption of myocardial iron in response to chelation therapy, with apical inferoseptum showing greater improvement as compared to the other segments [36]. LV GLS has the additional advantage of detecting myocardial dysfunction confined to specific areas of the myocardium, which may make it specifically suited for iron overload-related cardiomyopathy [12]. In our study, out of the 8 patients with abnormal GLS in one or more of the contributory echocardiographic views [AP4, AP3, or AP2 views] despite having normal mean GLS, one had reduced LVEF. Since the significance of this pattern of regional abnormality is not known at present, it was deemed appropriate to follow up these patients, although they were not considered to have LV dysfunction at present.

The major limitation of the study was its cross sectional design. The natural history of subclinical myocardial dysfunction in this cohort–whether it will resolve with time, or remain the same forever without manifesting clinically, or progress gradually to culminate in clinical heart failure- needs to be ascertained with long-term follow-up studies. However, it is indeed significant to find that a proportion of an apparently normal, relatively young ex-thalassaemic cohort, who have been receiving appropriate iron chelation therapy, harbour asymptomatic myocardial dysfunction.

As the ex-thalassaemic population is expected to achieve a normal life span, whether the subclinical myocardial dysfunction makes them more susceptible to subsequent unrelated cardiac morbidity in the future is another area of concern which needs to be addressed. Our study underlines the fact that close monitoring and follow-up of ex-thalassaemics for progressive myocardial dysfunction and clinical heart failure is warranted.

## Conclusion

There is a high prevalence of subclinical myocardial dysfunction in the ex-thalassaemic population, reiterating the need for close follow up of these patients. 2D speckle tracking echocardiography-derived LV global longitudinal strain is an effective tool in the evaluation of these patients, which should become a part of the follow-up even in asymptomatic patients, at least till iron overload is corrected.

## Supporting information

S1 Checklist *PLOS ONE* clinical studies checklist. (DOCX)

S1 Data set (XLSX)
